# Genetic and compositional analysis of biofilm formed by *Staphylococcus aureus* isolated from food contact surfaces

**DOI:** 10.3389/fmicb.2022.1001700

**Published:** 2022-12-02

**Authors:** María Guadalupe Avila-Novoa, Oscar Alberto Solis-Velazquez, Pedro Javier Guerrero-Medina, Jean-Pierre González-Gómez, Berenice González-Torres, Noemí Yolanda Velázquez-Suárez, Liliana Martínez-Chávez, Nanci Edid Martínez-Gonzáles, Lucia De la Cruz-Color, Luz María Ibarra-Velázquez, Marco Antonio Cardona-López, Miguel Ángel Robles-García, Melesio Gutiérrez-Lomelí

**Affiliations:** ^1^Centro de Investigación en Biotecnología Microbiana y Alimentaria, Departamento de Ciencias Básicas, Centro Universitario de la Ciénega, Universidad de Guadalajara, Ocotlán, Jalisco, Mexico; ^2^Laboratorio Nacional para la Investigación en Inocuidad Alimentaria (LANIIA), Centro de Investigación en Alimentación y Desarrollo, A.C. (CIAD), Culiacán, Sinaloa, Mexico; ^3^Laboratorio de Microbiología e Inocuidad de Alimentos, Departamento de Farmacología, Centro Universitario de Ciencias Exactas e Ingenierías, Universidad de Guadalajara, Guadalajara, Jalisco, Mexico

**Keywords:** *Staphylococcus aureus*, biofilms, extracellular matrix, food contact surface, genotypic characterization

## Abstract

**Introduction:**

*Staphylococcus aureus* is an important pathogen that can form biofilms on food contact surfaces (FCS) in the dairy industry, posing a serious food safety, and quality concern. Biofilm is a complex system, influenced by nutritional-related factors that regulate the synthesis of the components of the biofilm matrix. This study determines the prevalence of biofilm-associated genes and evaluates the development under different growth conditions and compositions of biofilms produced by *S. aureus*.

**Methods:**

Biofilms were developed in TSB, TSBG, TSBNaCl, and TSBGNaCl on stainless-steel (SS), with enumeration at 24 and 192 h visualized by epifluorescence and scanning electron microscopy (SEM). The composition of biofilms was determined using enzymatic and chemical treatments and confocal laser scanning microscopy (CLSM).

**Results and discussion:**

A total of 84 *S. aureus* (SA1–SA84) strains were collected from 293 dairy industry FCS (FCS-stainless steel [*n* = 183] and FCS-polypropylene [*n* = 110]) for this study. The isolates harbored the genes *sigB* (66%), *sar* (53%), *agrD* (52%), *clfB/clfA* (38%), *fnbA/fnbB* (20%), and *bap* (9.5%). 99. In particular, the biofilm formed by *bap*-positive *S. aureus* onto SS showed a high cell density in all culture media at 192 h in comparison with the biofilms formed at 24 h (*p* < 0.05). Epifluorescence microscopy and SEM revealed the metabolically active cells and the different stages of biofilm formation. CLSM analysis detected extracellular polymeric of *S. aureus* biofilms on SS, such as eDNA, proteins, and polysaccharides. Finally, the level of detachment on being treated with DNase I (44.7%) and NaIO 4(42.4%) was greater in the biofilms developed in TSB compared to culture medium supplemented with NaCl at 24 h; however, there was no significant difference when the culture medium was supplemented with glucose. In addition, after treatment with proteinase K, there was a lower level of biomass detachment (17.7%) of the biofilm developed in TSBNaCl (*p* < 0.05 at 24 h) compared to that in TSB, TSBG, and TSBGNaCl (33.6, 36.9, and 37.8%, respectively). These results represent a deep insight into the composition of *S. aureus* biofilms present in the dairy industry, which promotes the development of more efficient composition-specific disinfection strategies.

## Introduction

*Staphylococcus aureus* has various implications within nosocomial diseases and foodborne illnesses in terms of public health and economic effect. *S. aureus* has caused 1,681 illnesses and 86 hospitalizations reported in the foodborne-associated outbreaks in the United States (Dewey-Mattia et al., 2018); decreased animal production and milk production caused by clinical and subclinical mastitis in dairy animals; and increased use of antimicrobials for the treatment and prevention of mastitis and numerous diseases, including abscesses, septicemia, and pneumonia, which could lead to the emergence of antimicrobial resistance (Kumar et al., 2010; Akanbi et al., 2017; Abdi et al., 2018; Elsayed et al., 2019; Zhang et al., 2020). In addition, *S. aureus* can form biofilm and could be involved in 65–85% of microbial and chronic infections that are associated with biofilm formation reported by the National Institutes of Health (Flemming et al., 2016; Jamal et al., 2018).

*Staphylococcus aureus* can produce biofilm using different strategies, including (i) expression of the polysaccharide intercellular adhesin (PIA) by the *icaADBC* operon; (ii) release of extracellular DNA (eDNA); and (iii) expression of numerous surface proteins including MSCRAMMs (microbial surface components recognizing adhesive matrix molecules) (Archer et al., 2011; Dakheel et al., 2016). *S. aureus* produces a variety of MSCRAMMs such as fibronectin-binding proteins (FnBPs), *S. aureus* surface protein G (SasG), clumping factors A and B (ClfA, ClfB), the serine/aspartate-rich (Sdr) protein family, and biofilm-associated protein (Bap), which are protein components of the microbial surface that mediate the initial attachment to the surface proteins of host cells or binding to abiotic surfaces generate biofilms (Arciola et al., 2005; Renner and Weibel, 2011; Gutiérrez et al., 2012; Otto, 2018; Schllcher and Horswill, 2020).

Subsequently, the amounts of individual components of the extracellular matrix of S. *aureus* biofilms such as polysaccharides, glycoproteins, cell-surface-secreted bacterial proteinaceous adhesins, eDNA, and teichoic acids are influenced by different environmental conditions such as the culture medium, different *S. aureus* isolates, and interaction between different species and the surface (Flemming and Wingender, 2010; Schwartz et al., 2016; Singh et al., 2017; Liu et al., 2018; Sugimoto et al., 2018; Solis-Velazquez et al., 2021).

*Staphylococcus aureus* can produce biofilm in food processing environments and on equipment, including food contact surfaces (FCSs) (both food contact and non-food contact), pipelines, pasteurizers, and raw milk storage tanks, in the dairy industry (Gutiérrez et al., 2012; Di Ciccio et al., 2015; Avila-Novoa et al., 2018b). The persistence of biofilm in food processing environments is due to equipment designs that are difficult to clean and disinfect, ineffective cleaning of the food manufacturing environment, or interaction of antimicrobials and disinfectants with the extracellular matrix, decreasing their effectiveness, which has hindered strategies for the control of biofilms within the industry (Brooks and Flint, 2008; Bridier et al., 2015; Hall and Mah, 2017).

Biofilm is a potential source of direct and indirect contamination among food products and responsible for damaged equipment or drinking-water distribution, more expensive energy costs, and outbreaks (Møretrø and Langsrud, 2017; Alvarez-Ordóñez et al., 2019; Avila-Novoa et al., 2021; Carrascosa et al., 2021). Biofilms generate major food safety problems and economic losses for the food industry.

Hence, there is a need for knowledge about the factors that regulate the components in the extracellular matrix and the development or growth of biofilms, such as environmental conditions and phenotypic and genotypic characterization of biofilm-forming *S. aureus* that differ from those in planktonic conditions and which contribute to better adaptation of pathogens in a food processing environment, to establish control measures for the removal of biofilms. Therefore, the main objectives of this research were as follows: (i) to determine the prevalence of biofilm-associated genes in the *S. aureus* isolates and (ii) to provide useful data about the biofilm development under different growth conditions and the composition of biofilms formed by *S. aureus*.

## Materials and methods

### Bacterial strains

A total of 84 *S. aureus* strains were recovered from FCS in the dairy industry of Jalisco. In brief, 35.7% of enterotoxigenic *S. aureus* harbored 2–4 enterotoxin genes (*sea*, *seb*, *sec*, *sed*, *see*, *seh*, *sei*, and *sej*) (Avila-Novoa et al., 2018a) and 52.3% of the *S. aureus* contained the *icaADBC* gene that synthesizes PIA (Avila-Novoa et al., 2018b). Stocks were stored in tryptic soy broth (TSB; Becton Dickinson Bioxon, Le Pont de Claix, France) containing 30% glycerol at 80^°^C. Working cultures were maintained in TSB for 24 h at 37^°^C.

### Presence of *Staphylococcus aureus* adhesion and biofilm-related genes

Genomic DNA was extracted from *S. aureus* strains using a Bacteria DNA Preparation Kit (Jena Bioscience, Dortmund, Germany) according to the manufacturer’s instructions. All *S. aureus* strains were investigated for the detection of *clfB*, *clfA*, *fnbA*, *fnbB*, and *bap genes* by PCR using the protocol of Tang et al. (2013); *agrD*, *sar*, and *sigB* in the DNA were also determined (Kim et al., 2016). The amplification conditions used were as follows: 5 min at 95^°^C; 35 cycles of 40 s at 95^°^C, 50 s at different temperatures for different genes (Supplementary Table 3) and 50 s at 72^°^C; followed by a final extension of 10 min at 72^°^C. After that, the PCR products were electrophoresed on 1% agarose gel (UltraPure agarose, Invitrogen, Carlsbad, CA, USA), containing green gel loading buffer (Jena Bioscience, Dortmund, Germany) and visualized by transillumination under UV light (UVP, DigiDoc-It Darkroom, Upland, CA, USA). *S. aureus* ATCC 25923 was used as the positive control.

### Evaluation of cell viability and matrix characterization of biofilms under various environmental conditions

#### Surface preparation and quantification of biofilm formation

Stainless-steel (SS) coupons (AISI 316, 0.8 × 2.0 × 0.1 cm; CIMA Inoxidable, Jalisco, Mexico), prepared as described by Marques et al. (2007), were used as the surfaces for biofilms formation. In brief, the individual sterile SS coupons were introduced into a polypropylene tube (15 ml Centrifuge Tube, Corning CentriStar) containing 10 ml of TSB, TSB + 0.4% glucose (Golden Bell, Zapopan, México) (TSBG), TSB + 4% NaCl (Golden Bell, Zapopan, México) (TSBNaCl), or TSB + 0.4% glucose + 4% NaCl (TSBGNaCl) and then inoculated with 100 μl of the corresponding strain (∼10^8^ cfu/ml). Next, the polypropylene tubes were incubated at 37^°^C for 24 and 192 h, allowing the formation of biofilm. Bacterial enumeration of biofilms after incubation was conducted as previously described by Avila-Novoa et al. (2021). Three replicates were performed for each strain. *S. aureus* ATCC 25923 was used as the positive control.

#### Epifluorescence microscopy and scanning electron microscopy

After 24 and 192 h of incubation, the SS coupons were removed from the polypropylene test tubes containing 10 ml of TSB, TSBG, TSBNaCl, or TSBGNaCl, and the non-adhered cells were eliminated with PBS and vortexed for 10 s. In brief, cell viability was examined by staining cells with 5 (6)-carboxyfluorescein diacetate (CFDA, 10 μg/ml; Sigma-Aldrich, St. Louis, MO, USA) as described by Avila-Novoa et al. (2018b). Epifluorescence microscopy was performed using a Nikon Eclipse E400, a 100x oil immersion lens, and a blue excitation filter (BA 515 B-2A), at an emission wavelength of 450–490 nm. Scanning electron microscopy (SEM) was performed on the SS coupons after 24 and 192 h of incubation using the protocols described by Borucki et al. (2003) and Fratesi et al. (2004). Biofilms were observed using a TESCAN Mira3 LMU scanning electron microscope (Tezcan, Prague, Czech Republic).

### Evaluation of biofilms with composition by confocal laser scanning microscopy and detachment assays

#### Confocal laser scanning microscopy

After incubation at 37^°^C for 24 and 192 h, the SS coupons were thoroughly rinsed with PBS and vortexed for 10 s to eliminate the non-adhered cells. Then, for observation, the components of the biofilm were exposed to the following three dyes: (i) SYTO 9^®^ Green-Fluorescent Nucleic Acid Stain (Invitrogen, Eugene, OR, USA) (excitation, 476 nm; emission, 500–520 nm) which stains nucleic acids, (ii) FilmTracer™ SYPRO^®^ Ruby Biofilm Matrix Stain (Invitrogen, Eugene, OR, USA) (excitation, 405 nm; emission, 655–755 nm) which labels most classes of proteins, and (iii) WGA, wheat germ agglutinin conjugated with Oregon Green (Invitrogen, Eugene, OR, USA) (excitation, 459 nm; emission, 505–540 nm) which stains *N*-acetyl-D-glucosamine residues (Oniciuc et al., 2016). Subsequently, microscopic observation and image analysis of biofilms were performed with a Zeiss LSM 700 confocal laser scanning microscope (Carl Zeiss, Germany) and ZEN 2009 V 5.5 Software (Carl Zeiss^®^, Jena, Germany).

#### Matrix characterization

Biofilm detachment assays were carried out as described by Oniciuc et al. (2016) and Avila-Novoa et al. (2021). Mature biofilms were treated with (i) proteinase K (PROMEGA, Madison, WI, USA) (0.1 mg ml^–1^ in 20 mM Tris-HCl: 1 mM CaCl_2_), (ii) 40 mM NaIO_4_ in double-distilled H_2_O, or (iii) 0.5 mg ml^–1^ DNase I (Roche, Mannheim, Germany) in 5 mM MgCl_2_, for 2 and 24 h at 37^°^C. Previously, the mature biofilms were cultivated in TSB, TSBG, TSBNaCl, or TSBGNaCl (37^°^C for 192 h) and subsequently washed with 0.9% NaCl for treatment. Biomass quantification was performed by measuring the optical density (OD) at 492 nm (OD_492_) using a Multiskan FC (Thermo Fisher Scientific Inc., Madison, WI, USA). All experiments were performed in triplicate.

### Statistical analysis

All experiments were evaluated using analysis of variance (ANOVA), followed by a least significant difference (LDS) test, in the Statgraphics Centurion XVI software program (StatPoint Technologies, Inc., Warrenton, VA, USA). Values of *p* < 0.05 were considered statistically significant.

## Results

**FIGURE 1:**
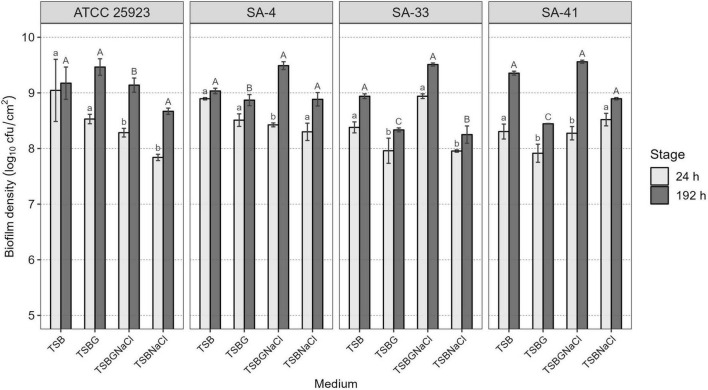
Cell density of *S. aureus* biofilms developed in different enriched media at 24 and 192 h of maturity. Different lowercase letters indicate significant differences between S. aureus strains on the same growth medium at 24 h of development and uppercase letters at 192 h, according to the Tukey test (*p* < 0.05). TSB, Tryptic soy broth; TSBG, TSB + 0.4% Glucose; TSBNaCl, TSB + 4% NaCl; TSBGNaCl, TSB + 0.4% Glucose + 4% NaCl.

**FIGURE 2:**
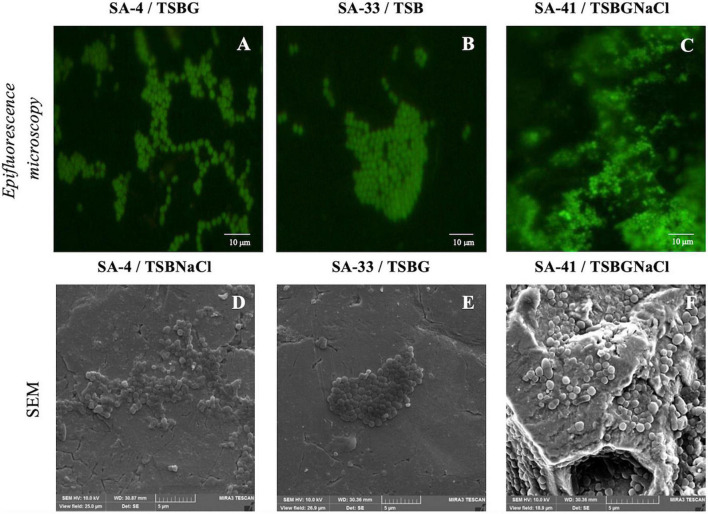
Biofilms of isolates *S. aureus* [SA-4 **(A,D)**, SA-33 **(B,E)**, and SA-41 **(C,F)**] from food contact surfaces (FCS). Biofilms were developed on stainless steel through 192 h of incubation in different medium at 37^°^C and visualized with epifluorescence microscopy (top row) and SEM (bottom row). SA, *S. aureus*; TSB, Tryptic soy broth; TSBG, TSB + 0.4% Glucose; TSBNaCl, TSB + 4% NaCl; TSBGNaCl, TSB + 0.4% Glucose + 4% NaCl.

Of all isolates, 66% (56/84) harbored *sigB*, 53% (45/84) *sar*, 52% (44/84) *agrD*, 38% (32/84) *clfB*, 38% (32/84) *clfA*, 20% (17/84) f*nbA*, 20% (17/84) f*nbB*, and 9.5% (8/84) *bap*. Of the 22 isolates, *agrD*, *sigB*, and *sar* were detected in 26%, and *clfA*, *clfB*, *fnbA*, and *fnbB* were detected in 13% (Table 1). Subsequently, a selection of three strains of *S. aureus* (SA-4, SA-33, SA-41) was based on the genotypic and phenotypic characteristics associated with the formation of biofilm, in addition to the risks associated with the consumer by the detection of enterotoxigenic genes involved in food poisoning in previous publications (Avila-Novoa et al., 2018a,b; Table 2). All the tested microorganisms (*bap*-positive *S. aureus* [SA-4, SA-33, SA-41]) showed a strong ability to develop biofilms in TSB (8.30–9.04 log_10_ cfu/cm^2^), TSBG (7.91–8.53 log_10_ cfu/cm^2^), and TSBGNaCl (8.28–8.94 log_10_ cfu/cm^2^), compared to TSBNaCl (7.84–8.52 log_10_ cfu/cm^2^; *p* < 0.05) at 24 h; however, the development of the biofilm *S. aureus* was favored at 192 h (*p* < 0.05) (Figure 1). Generally, *S. aureus* formed a higher cellular density biofilm in TSBGNaCl (9.14–9.56 log_10_ cfu/cm^2^) in comparison with TSBNaCl (8.25–8.89 log_10_ cfu/cm^2^; *p* < 0.05) and TSBG (8.34–9.47 log_10_ cfu/cm^2^; *p* < 0.05) at 192 h. Besides that, *S. aureus* biofilm had a lower biomass biofilm in TSBNaCl in comparison with TSB (8.94–9.35 log_10_ cfu/cm^2^; *p* < 0.05); there was no difference between TSBNaCl and TSBG (*p* > 0.05), TSBG and TSB (*p* > 0.05), and TSB and TSBGNaCl (*p* > 0.05) at 192 h (Figure 1). Furthermore, there was no significant difference in the biofilm formation capacity of SA-4, SA-33, SA-41, and *S. aureus* ATCC 25923 in unsupplemented TSB (24–192 h; *p >* 0.05). A comparison of supplemented media showed that TSBG decreased the cell density (<1 log_10_ cfu/cm^2^) of SA-33 and SA-41 (TSBG; *p <* 0.05); TSBGNaCl decreased that of SA-4, SA-41, and *S. aureus* ATCC 25923 (*p <* 0.05), and TSBNaCl that of SA-33 and *S. aureus* ATCC 25923 (*p <* 0.05) at 24 h. The cellular density of biofilm of SA-4, SA-33, and SA-41 on TSBG (*p <* 0.05) and that of SA-33 on TSBNaCl (*p <* 0.05) was decreased compared to the cell density of SA-41 which increased (>1 log_10_ cfu/cm^2^) on TSBGNaCl (*p <* 0.05) at 192 h. Epifluorescence micrographs showed adhered-embedded cells in possible extracellular polymeric substances (EPS) and microcolonies made up of metabolically active cells (Figures 2A–C). In addition, SEM analysis showed the different stages of biofilm formation with adhered cells, forming microcolonies embedded in EPS, or maturation and dispersal of cells, highlighting the rheological structure of the biofilm (Figures 2D–F).

**TABLE 1:** Frequency of *agrD*, *sar*, *sigB*, and *adhesin genes* in *Staphylococcus aureus* isolates from food contact surfaces (FCSs) in the dairy industry.

Genotype	No. (%) of *S. aureus* strain isolated
*clfA-clfB-fnbpA-fnbpB-bap-agrD-sigB-sar*	4 (4.76)
*clfA-clfB-fnbpA-fnbpB-agrD-sigB-sar*	1 (1.19)
*clfA-clfB-fnbpA-fnbpB-sigB-sar*	1 (1.19)
*clfA-clfB-fnbpA-fnbpB-agrD-sar*	1 (1.19)
*fnbpA-fnbpB-agrD-sigB-sar*	4 (4.76)
*clfA-clfB-fnbpA-fnbpB-agrD*	1 (1.19)
*clfA-clfB-fnbpA-fnbpB-sar*	1 (1.19)
*clfA-clfB-fnbpA-fnbpB-sigB*	1 (1.19)
*clfA-clfB-fnbpA-fnbpB-bap*	1 (1.19)
*clfA-clfB-agrD-sigB-sar*	1 (1.19)
*clfA-clfB-bap-agrD-sigB*	1 (1.19)
*clfA-clfB-agrD-sigB*	6 (7.14)
*agrD-sigB-sarA-bap*	1 (1.19)
*clfA-clfB-sar*	1 (1.19)
*clfA-clfB-agrD*	2 (2.38)
*clfA-clfB-sigB*	4 (4.76)
*agrD-sigB-sar*	12 (14.28)
*sigB-sarA-bap*	1 (1.19)
*fnbpA-fnbpB-agrD*	1 (1.19)
*fnbpA-fnbpB-sigB*	1 (1.19)
*clfA-clfB*	6 (7.14)
*agrD-sigB*	8 (9.52)
*sigB-sar*	7 (8.33)
*AgrD*	5 (5.95)
*SigB*	3 (3.57)
*Sar*	2 (2.38)

**TABLE 2:** Characteristics associated with biofilm formation of *Staphylococcus aureus* in this study.

Bacterial strain	^a^SE’s/ ^b^*icaADBC*	Genotypes biofilm related							
		*clfA*	*clfB*	*fnbpA*	*fnbpB*	*bap*	*agrD*	*sigB*	*sar*
SA-4	*sec* + *sed* + *seg* + *sej/icaADBC*	+	+	+	+	+	+	+	+
SA-33	*sej/icaADBC*	−	+	+	+	+	+	+	+
SA-41	–/icaAD	−	+	+	+	+	+	+	+

^a^, ^b^Avila-Novoa et al. (2018a,b).

**FIGURE 3:**
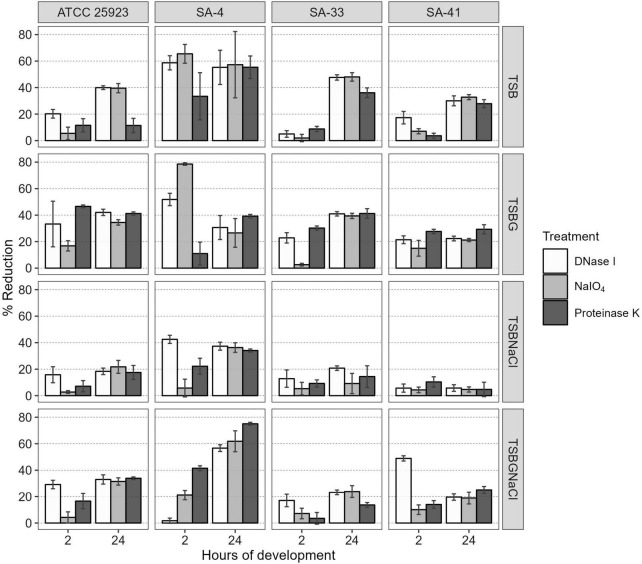
Percentage reduction of *S. aureus* biofilms after treatment with DNase I, NaIO4, or proteinase K at 2 and 24 h of maturity. SA, *S. aureus*; TSB, Tryptic soy broth; TSBG, TSB + 0.4% Glucose; TSBNaCl, TSB + 4% NaCl; TSBGNaCl, TSB + 0.4% Glucose + 4% NaCl.

**FIGURE 4:**
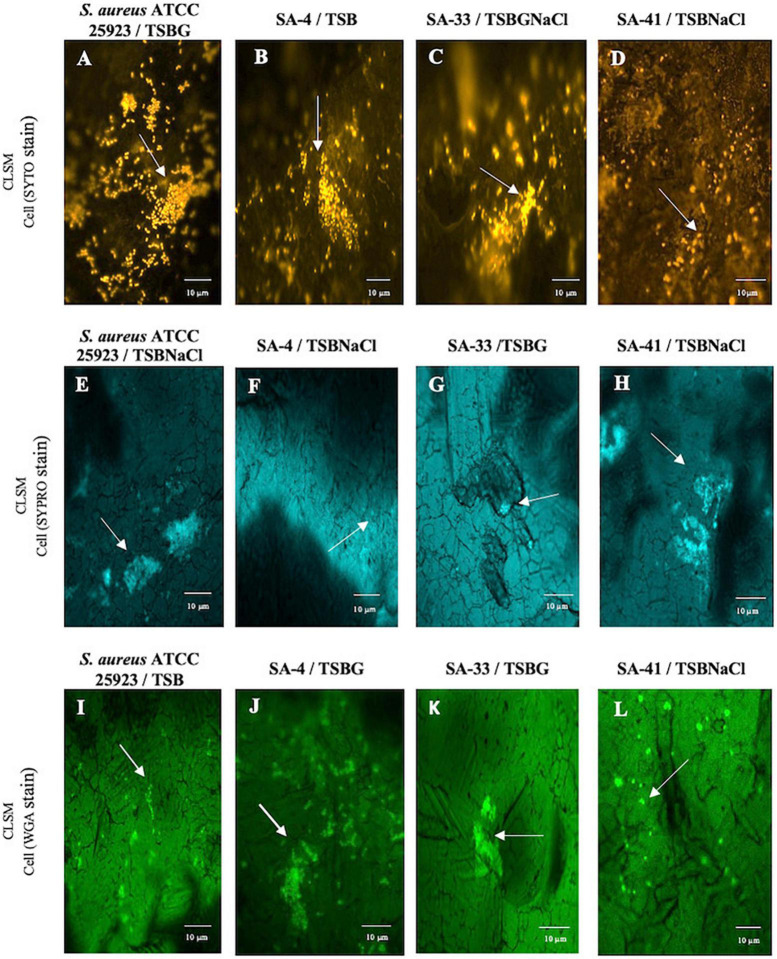
Biofilm matrix structures obtained from CLSM observation of *S. aureus* ATCC 25923 **(A,E,I)**, SA-4 **(B,F,J)**, SA-33 **(C,G,K)**, and SA-41 **(D,H,L)** isolates from FCS on stainless steel through h of incubation in 37^°^C for 192 h in different medium. SA, *S. aureus*; TSB, Tryptic soy broth; TSBG, TSB + 0.4% Glucose; TSBNaCl, TSB + 4% NaCl; TSBGNaCl, TSB + 0.4% Glucose + 4% NaCl.

Additionally, the components of the SA-4, SA-33, and SA-41 biofilms were determined by the detachment of the biofilm according to the medium in which it was developed (Figure 3). The level of the detachment of the biofilms developed after the treatments with NaIO_4_, DNase I, and proteinase *K* with an exposure time of 2 h was lower compared to that at 24 h (*p* < *0.05*). After treatment with NaIO_4_ and proteinase *K*, there was a lower level of the detachment of the biomass of the biofilm developed in TSBNaCl (*p* < 0.05 at 2 h) compared to TSBG; however, there was no significant difference after treatment with DNase I in the biofilms developed in TSB, TSBG, TSBNaCl, and TSBGNaCl (*p* > 0.05 at 2 h). In general, after treatment with DNase I, the level of biomass detachment was greater (44.7%) of the biofilm developed in TSB compared to TSBNaCl and TSBGNaCl (20.5 and 33.1%, respectively) (*p* < 0.05 at 24 h). The effects with NaIO_4_ was a greater level of biomass detachment (42.4%) of the biofilm developed in TSB compared to TSBNaCl (17.9%; *p* < 0.05 at 24 h); however, there was a significant difference in detached biofilm biomass generated between TSBG and TSBNaCl (*p* < 0.05 at 24 h) and TSBNaCl and TSBGNaCl (*p* < 0.05 at 24 h). In addition, after treatment with proteinase *K*, there was a lower level of biomass detachment (17.7%) of the biofilm developed in TSBNaCl (*p* < 0.05 at 24 h) compared to that in TSB, TSBG, and TSBGNaCl (33.6, 36.9, and 37.8%, respectively). SA-33 and SA-41 showed less detachment of biofilm biomass after the treatments with NaIO_4_, DNase I, and proteinase *K* compared to SA-4 (*p* < 0.05, at 2–24 h). Confocal laser scanning microscopy (CLSM) analysis in conjugation with three different fluorescent dyes was used to observe the biofilm production of *S. aureus*, and the results indicated that these biofilms were composed of bacterial cells and EPS. Biofilm matrices of SA-4, SA-33, and SA-41 were formed by different *S. aureus* and EPS such as eDNA (Figures 4A–D), proteins (Figures 4E–H), and polysaccharides adhesin (Figures 4I–L).

## Discussion

*Staphylococcus aureus* can generate biofilms on FCS within the dairy industry, affecting the quality and safety of food products. In brief, this pathogen causes outbreaks of foodborne illnesses associated with the consumption of milk and dairy products, and cow clinical and subclinical mastitis, which leads to huge economic losses.

In this study, the prevalence of genes encoding for the adhesion factors revealed 20% for (*fnbA/fnbB*) and 38% for (*clfA/clfB*) in the 84 *S. aureus* strains recovered from FCS in the dairy industry of Jalisco (Avila-Novoa et al., 2018a). Similar observations have also been reported by other investigators (Tang et al., 2013; Gogoi-Tiwari et al., 2015; Azara et al., 2017; Azmi et al., 2019; Liu et al., 2020; Zaatout et al., 2020) who reported similar percentages (*clfA* [2.3–41.6%], *clfB* [4.6%], *fnbA* [0–54.5%], and *fnbB* [1.3–68.7%]) for the prevalence of some of the adhesion genes. Also, Azara et al. (2017); Vergara et al. (2017), and Zhang et al. (2020) found no evidence of the *fnbA* or *fnbB* genes in *S. aureus*. Additionally, 9.5% of the *S. aureus* isolates had *bap* which has been associated with *S. aureus* of bovine mastitis origin and with *ica*-independent biofilm formation (Arciola et al., 2001; O’Gara, 2007; Archer et al., 2011; Salgado-Ruiz et al., 2015; Aslantaş and Demir, 2016).

In contrast, a considerably greater prevalence of *clfA* (50–100%), *clfB* (80.2–100%), *fnbA* (72.8–100%), and *fnbB* (80.3–100%) genes have been found in *S. aureus* isolates collected from milk samples from cows with clinical and subclinical mastitis, pasteurized milk, chicken, food poisoning outbreaks, pork, and slaughtered goats (Tang et al., 2013; Aslantaş and Demir, 2016; Pereyra et al., 2016; Azara et al., 2017; Vergara et al., 2017; Dai et al., 2019; Liu et al., 2020; Zhang et al., 2020). Also, the *bap* gene was not detected in isolates of S. *aureus* by Tang et al. (2013); Pereyra et al. (2016), and Liu et al. (2020).

This suggests that a variety of virulence factors such as ClfA, ClfB, FnbA, FnbB, and Bap are involved in the initial attachment to the surface proteins of host cells and colonization of the mammary gland by *S. aureus*. Besides, binding to abiotic surfaces such as FCS, these virulence factors are involved in the initial stage of biofilm formation or with components inside EPS to give biofilm stability.

Nevertheless, the variation in the prevalence of *S. aureus* virulence factors in this study could be associated with the genetic diversity of strains, epidemiological factors where different sources or mechanisms of contamination in the food processing environment are involved in each of the developed countries, and the source and sizes of samples or their geographic locations. Some virulence factors of *S. aureus* are encoded in plasmids or phages which can be transferred between bacteria by horizontal gene transfer where the exchange and transfer of genes are facilitated by biofilm formation (Fueyo et al., 2005; Beceiro et al., 2013; Derakhshan et al., 2021).

Biofilm formation by *S. aureus* not only involves the *icaADBC* operon, *agr* locus, and quorum-sensing mechanisms (QS) but also other biofilm-associated genes such as *arlRS*, *bap*, *hla*, *rbf*, *sar*, *sigB*, *tcaR*, and *trap* (Tsang et al., 2008; Ciftci et al., 2009; Aslantaş and Demir, 2016; Kim et al., 2016; Sankar Ganesh et al., 2019). Following other studies (Kim et al., 2016; Avila-Novoa et al., 2021), we found that the presence of *sigB*, *sar*, and *agrD* genes was associated with the regulation and formation of biofilms; also, *agr* regulates the production of biofilms, including the detachment of biofilm, and then the expression of virulence-associated gene expression helps in dissemination of *S. aureus* (Boles and Horswill, 2008; Paharik and Horswill, 2016). However, the absence of regulators such as *sigB*, *agrD*, and *sar* in isolates *S. aureus* could be for the presence of other regulators such as MgrA and ArIRS have also been linked to biofilm formation. Hence, three strains of different *bap*-positive *S. aureus* were investigated for evaluation of cell viability and matrix characterization of biofilms under various environmental conditions. In this study, we also found that the cell density of *bap*-positive *S. aureus* biofilms is higher in medium supplements with glucose (8.34–9.47 log_10_ cfu/cm^2^ in TSBG) or glucose and NaCl (9.14–9.56 log_10_ cfu/cm^2^ in TSBGNaCl) at 192 h (*p* < 0.05). Besides, the cell density of a biofilm is lower in TSBNaCl (24–192 h; *p* < 0.05) (Figure 1). The observations emphasize the fact that the ability to form biofilm is complex when using medium supplements (TSBG, TSBNaCl, and TSBGNaCl) compared to TSB. Similar observations have also been reported by other investigators (O’Neill et al., 2007; Croes et al., 2009; Srey et al., 2013): the evidence of glucose or NaCl induce biofilm formation for *S. aureus*. This could be associated with the fact that the supplements favor the pre-conditioning of the surface and irreversible adhesion for the formation of the biofilm and are associated with the genotypic characteristics of *S. aureus* (Table 2). Likewise, Cucarella et al. (2001) reported that *bap*-positive *S. aureus* can form biofilm even though its *icaADBC* operon was disrupted. Also, pre-conditioning influences the chemical and physical properties of the substrate/fluid interface making it a more favorable environment for bacterial adhesion (Chmielewski and Frank, 2003; Lorite et al., 2011).

The presence of high concentrations of glucose in the medium decreases pH due to catabolism; however, this represses *agr-*locus favoring the biofilm formation of *S. aureus* (O’Neill et al., 2007; McCarthy et al., 2015; Paharik and Horswill, 2016). Taglialegna et al. (2016) determined the expression of *bap* and the formation of the biofilm of *S. aureus* V329 (Bap-positive) in LB-glucose, concluding that Bap promotes the aggregation de Bap-positive strains and the development of the biofilm, where the pH decreases (pH < 5) due to the growth of *S. aureus* in the LB-glucose medium. Likewise, Sugimoto et al. (2018) argue that low pH limits the production of extracellular proteases, inducing the association of surface proteins in the extracellular matrix and promoting the formation of biofilms. Lade et al. (2019) determined differences in biofilm formation when there are NaCl supplements (1–2%) to the TSB and they associate it with the loose attachment of *S. aureus* biofilms to the surface due to an excess of NaCl. There is an association between methicillin-sensitive *S. aureus* (MSSA) or methicillin-resistant *S. aureus* (MSRA) and *ica*-dependent biofilm. O’Neill et al. (2007) determined that NaCl-induced biofilm development was significantly more prevalent in MSSA clinical isolates compared with MRSA; however, various external signals, such as pH, incubation temperatures, ingredients composition (glucose, sodium chloride, ethanol, caseins, serum albumin, fibrin, and dilution rate of media), and CO_2_, (Kumar et al., 2017; Miao et al., 2017; Solis-Velazquez et al., 2021), can alter the regulation and the expression of biofilm-associated genes and regulators and/or biofilm development.

Overall, biofilm *S. aureus* matrix presented greater detachment of biofilm after DNase I (44.7%) and NaIO_4_ (42.4%) treatment in TSB as compared to low detachment of biofilm in TSBNaCl (*p* < 0.05 at 24 h) (Figure 3). Dakheel et al. (2016) and Oniciuc et al. (2016) reported similar percentages of polysaccharide levels (20–52%) for *S. aureus* isolates from different systemic infections, dairy products, fish and fish products, and meat and meat products. In contrast, other researchers, Fredheim et al. (2009) and Solis-Velazquez et al. (2021), have reported low biofilm detachment after DNase I treatment in *S. epidermidis* (20%) and *S. aureus* (7.95%).

In addition, after treatment with proteinase *K*, there was a lower level of biomass detachment (17.7%) of the biofilm developed in TSBNaCl (*p* < 0.05 at 24 h) compared to that in TSB, TSBG, and TSBGNaCl (33.6, 36.9, and 37.8, respectively) in this study. A similar observation was also reported by Dakheel et al. (2016) and Oniciuc et al. (2016), who showed detachment of biofilm for *S. aureus* strains isolated from isolates of different systemic infections and food sources after proteinase *K* treatment (39–70%). Likewise, Shukla and Rao (2017) showed that *bap*-positive *S. aureus* V329 and other *S. aureus* (SA7, SA10, SA33, and SA352) bovine mastitis isolates had a biofilm detachment of about 60–84% after proteinase *K* treatment. In contrast, Fredheim et al. (2009) and Solis-Velazquez et al. (2021) have reported low biofilm detachment after proteinase *K* treatment in *S. epidermidis* (10%) and *S. aureus* (12.5%). However, proteinase *K* treatment did not affect the *bap*-mutant *S. aureus* M556 or *bap*-negative *S. aureus* biofilm (Shukla and Rao, 2013, 2017).

Our data showed that the different levels of polysaccharide, proteins, and eDNA after treatments may be associated with the expression or regulation of the *icaADBC* operon, *agr-*locus, etc., in *S. aureus*, biofilm age, environmental factors, and conditions of treatment enzymatic such as concentration, period of contact, type surface etc. Bai et al. (2021) argue that the surface materials, growth conditions, and biofilm maturity affected the composition of complex extracellular matrixes (ECMs) of *S. aureus.* Clearly, eDNA is one of the main components of the biofilm in this study; however, eDNA plays several roles such as bacterium surface adhesion by modulation of charge and hydrophobicity interactions between the bacteria and the abiotic surface (Nguyen et al., 2016) and chelates divalent cations, which triggers a genetic response to increase pathogenicity and resistance to antimicrobials (Okshevsky and Meyer, 2015). Abdallah et al. (2014) argue that the increase in the biofilm age also promoted increases in the proteins and carbohydrates in the matrix of the *S. aureus* biofilm.

NaIO_4_ can modify PIA/poly N-acetylglucosamine (PNGA) polymer chains by cleaving C3-C4 bonds in exopolysaccharide residues and oxidizing carbons to produce vicinal hydroxyl groups (Chaignon et al., 2007). Nevertheless, the low or high level of exopolysaccharides after NaIO_4_ treatment in MRSA may be due to differences both in the amount of O-linked acetates with succinate and acetylation levels of amino groups (Spiliopoulou et al., 2012; Dakheel et al., 2016). Besides, polysaccharides are not the only component within the biofilm matrix, there are other components such as eDNA, proteins, and lipids, that interact with each other and can affect detachment. Likewise, Oniciuc et al. (2016) proved that protein-based matrices are of prime importance for the structure of biofilms formed by *S. aureus* strain isolates from food sources; however, the biofilms are composed of different types of proteins, which may vary from one *S. aureus* strain to another (Chaignon et al., 2007). The results obtained by CLSM allowed visual analysis of the concurrent distribution of eDNA, protein, and polysaccharide components within the biofilms and SEM enable observation of the biofilm architecture (EPS and embedded bacterial cells) (Figures 2, 4). The heterogeneity of the biofilm matrix limits the effect of the biocides and/or by quenching their action (Araújo et al., 2013; Lutskiy et al., 2015). However, in this study, it is suggested that proteinase *K* and DNase I allow the dispersion of the biofilm, or they could be facilitating the penetration of other biocides into the biofilm. The proteinase *K* has a synergistic effect when associated with antibiotics, and DNase I has anti-biofilm activity against *S. aureus* biofilm (Kaplan et al., 2012; Shukla and Rao, 2013, 2017). Consequently, it is very urgent and significant to establish control strategies and prevention methods in food industries where they have incorporated the use of two or several successive treatments that may be necessary for a sufficient remotion of biofilm produced by *S. aureus.*

## Conclusion

Most of the *S. aureus* strains isolated from FCS in the dairy industry of Jalisco harbored virulence-associated genes, and in addition, they carried genes associated with the formation of biofilms. The biofilms formed with the selected strains showed different compositions of EPS. Our study showed that the proportion components that make up the extracellular matrix are associated with factors such as culture media and genetic characteristics of the *S. aureus* isolates. Determining the virulence potential of *S. aureus* is important in terms of public health, as is risk identification in milk and dairy products because they provide critical information for microbiological and chemical risk assessment.

## Data availability statement

The original contributions presented in this study are included in the article/Supplementary material, further inquiries can be directed to the corresponding author.

## Author contributions

MA-N and MG-L: conceptualization, resources and funding acquisition, supervision, and project administration. OS-V, J-PG-G, and BG-T: methodology and investigation. NV-S, LM-C, NM-G, and LD: validation and formal analysis. LI-V, MC-L, and MR-G: original draft preparation. MA-N, PG-M, and MG-L: writing—review and editing. All authors have read and agreed to the published version of the manuscript.
