# Supplementary material for: Genetic and compositional analysis of biofilm formed by Staphylococcus aureus isolated from food contact surfaces
Source: Front Microbiol. 2022 Dec 2;13:1001700. doi: 10.3389/fmicb.2022.1001700 (PMC9755592; doi:10.3389/fmicb.2022.1001700)
Supplement: Supplementary file 1 [file Table_1.docx]

Table 3 – Supplementary document.

Table 3. Primers for amplification of *S. aureus* biofilm-associated genes

| **Primers** | **Length (bp)** | **Sequence 5’—3’** | | **Temperature** | **Reference** |
| --- | --- | --- | --- | --- | --- |
| *fnbpA* | 952 | Forward | 5’-TCC GCC GAA CAA CAT ACC -3’ | 54 ºC | Tang et al. (2013) |
|  |  | Reverse | 5’-TCA AGC ACA AGG ACC AAT -3’ |  |  |
| *fnbpB* | 452 | Forward | 5’-TCT GCG TTA TGA GGA TTT -3’ | 54 ºC | Tang et al. (2013) |
|  |  | Reverse | 5’-ACA GTA GAG GAA AGT GGG-3’ |  |  |
| *bap* | 873 | Forward | 5’-GAG CCA AGA CAA AGG TGA AG -3’ | 58 ºC | Tang et al. (2013) |
|  |  | Reverse | 5’-GTA GCC ATA GCA CGG AAC AT -3’ |  |  |
| *clfA* | 796 | Forward | 5’-AGT ACC AAA TGA GGC TGT TC -3’ | 56 ºC | Tang et al. (2013) |
|  |  | Reverse | 5’-AAA TGC TAC TTC GTT GTC CC-3’ |  |  |
| *clfB* | 968 | Forward | 5’-CAC TTA CTT TAC CGC TAC TTT C-3’ | 57 ºC | Tang et al. (2013) |
|  |  | Reverse | 5’-AAC GAG CAA TAC CAC TAC AAC AG -3’ |  |  |
| *agrD* | 307 | Forward | 5’-CAT TCC TGT GCG ACT TAT TAA ACG-3’ | 56 ºC | Kim et al. (2016) |
|  |  | Reverse | 5’-CGT GTA ATT GTG TAA ATT CTT TTG C-3’ |  |  |
| *sar* | 867 | Forward | 5’-CGG TAC CGT TGA TTT GGG TAG TAT GC-3’ | 55 ºC | Kim et al. (2016) |
|  |  | Reverse | 5’-TTG CCA TGG TTA AAA CCT CCC-3’ |  |  |
| *sigB* | 937 | Forward | 5’-CGG ATC CGG TGT GAC AAT CAG TAT GAC-3’ | 55 ºC | Kim et al. (2016) |
|  |  | Reverse | 5’-CGG AAT TCG CGA CAT TTA TGT GGA TAC AC-3’ |  |  |
